# Characteristics of young and lower functioning patients following total knee arthroplasty: a retrospective study

**DOI:** 10.1186/s12891-019-2817-4

**Published:** 2019-10-27

**Authors:** Jesse C. Christensen, Andrew J. Kittelson, Brian J. Loyd, Michael A. Himawan, Charles A. Thigpen, Jennifer E. Stevens-Lapsley

**Affiliations:** 1 Department of Physical Medicine and Rehabilitation, Eastern Colorado Geriatric Research Education and Clinical Center, 13001 E. 17th Pl, Aurora, CO 80045 USA; 2 Department of Physical Medicine and Rehabilitation, University of Colorado, Aurora, CO USA; 3 Department of Physical Therapy and Athletic Training, University of Utah, Salt Lake City, UT USA; 4 ATI Physical Therapy, Greenville, SC USA

**Keywords:** Total knee arthroplasty, Clinical outcomes, Subgrouping, Self-report, Physical performance

## Abstract

**Background:**

Rates of total knee arthroplasty (TKA) procedures in younger, more medically complex patients have dramatically increased over the last several decades. No study has examined categorization of lower and higher functioning subgroups within the TKA patient population. Our study aimed to determine preoperative characteristics of younger patients who are lower functioning following TKA.

**Methods:**

Patients were categorized into higher and lower functioning subgroups defined using a median split of 1) postoperative Timed Up and Go (TUG) test times and 2) Western Ontario and McMaster Universities Osteoarthritis Index (WOMAC) physical function subscale scores. A split in age (65 years) was used to further classify patients into four categories: younger lower functioning, younger higher functioning, older lower functioning and older higher functioning. Measures from preoperative domains of health, psychological, physical performance and pain severity were examined for between-group differences.

**Results:**

Comparing mean values, the younger lower functioning subgroup using the TUG had significantly weaker knee extensor, slower gait speed, higher body mass index and greater pain compared to other subgroups. The younger lower functioning subgroup using the WOMAC physical function subscale demonstrated higher pain levels and Coping Strategies Questionnaire-Catastrophizing Subscale scores compared to the older lower functioning subgroup.

**Conclusions:**

Poorer preoperative physical performance and pain severity appear to have the largest influence on early postoperative TKA recovery in younger lower functioning patients relative to both younger and older higher functioning patients.

## Background

Total knee arthroplasty (TKA) is the most common and effective treatment for enhancing quality of life and alleviating pain associated with knee arthritis in aging adults [1, 2]. As the demand for TKA procedures continues to increase, so has the diversity of the patient population electing to undergo surgery. Historically, TKA has been viewed as a surgical procedure for older adults suffering with chronic knee pain due to end-stage arthritis. However, rates of TKA procedures in younger, more medically complex patients have dramatically increased over the last several decades [3–5]. Alarmingly, a growing number of younger patients report less satisfaction with their functional ability relative to older counterparts following surgery [4, 6–8].

Studies have shown 75–90% of patients are generally satisfied with the postoperative outcome of pain relief and functional improvement [9–11]. However, as high as 25% of patients report residual symptoms that affect their functional ability [9, 12] with the rate of dissatisfaction increasing to 34% in younger adults [8]. Few studies have investigated why some younger patients respond unfavorably to TKA. Despite increasing rates of surgery in younger, less healthy patients, these patients are often excluded from research participation [4, 6–8]. Studies have also shown preoperative characteristics (e.g. health, psychological, physical performance and pain intensity status) have a predictive influence on postoperative recovery in relatively older adults (≥ 65 years) following TKA, but it remains unclear if the same characteristics are present in younger lower functioning patients. Identifying preoperative characteristics for poor TKA outcomes could allow for improved care for this vulnerable and growing patient subgroup.

Furthermore, it is unknown whether the factors that predispose patients to a poor self-report of function are similar or different to the factors that predispose patients to a poor outcome measured by physical performance tests. Both self-report and physical performance measures with TKA are important to include when assessing characteristics, knowing patient’s perception of improved function contrasts with objective physical performance findings [13]. Studies commonly investigate self-reports of functioning [14], while physical performance measures are less frequently captured. However, both self-report and physical performance measures are needed to fully assess the domain of physical functioning, as they often provide discordant information [13]. This is especially true in the early postoperative period when patients perceive improvements in functioning that are not corroborated with physical performance findings [13, 15].

Therefore, the purpose of this study was to identify preoperative characteristics of a group of patients who are both relatively young and lower functioning following TKA surgery, to determine whether and how this group differs from those with a more typical recovery response postoperatively. We hypothesized that, regardless of the operational definition, the younger lower functioning subgroup would differ from other groups preoperatively in terms of poorer health status, lower psychological status, worse physical performance and higher pain intensity.

## Methods

### Study design and participants

An observational cohort study was conducted on a convenience sample of patients undergoing a primary unilateral TKA between January 2013 and August 2015 who met all eligibility requirements. Inclusion criteria were patients 40 through 90 years of age, completed a postoperative functional assessment [Timed Up and Go (TUG) and/or Western Ontario and McMaster Universities Osteoarthritis Index (WOMAC) physical function subscale] and underwent an uncomplicated primary unilateral TKA. Exclusion criteria included uni-compartmental knee arthroplasty, revision knee arthroplasty or scheduled for a staged TKA procedure (underwent ipsilateral and contralateral TKA < 10-weeks between procedures). Clinical data was extracted from health records at three sites within the Assessment Technologies Inc. (ATI) Physical Therapy clinics (Greenville, SC, USA) that were collected during routine physical therapy treatment visits. Data were extracted from existing health records to ensure an adequate representation of patients commonly excluded from research participation. The postoperative physical therapy regime was standardized across clinic locations and therapists. Phase 1 (0 to 4 weeks) of the physical therapy protocol consisted of passive, active-assist and active range of motion exercises, stationary bicycling, muscle-activation exercises and gait training. Phase 2 (4 to 8 weeks) focused on progressive range of motion and flexibility, muscle strengthening, neuromuscular control and functional exercises. Phase 3 (8 to12 weeks) focused on restoring optimal range of motion, muscle strengthening and progressing to higher-level recreational activities. All data was collected in part of standard clinical care and no informed consent was obtained. The Institutional Review Board at University of Colorado Denver (COMIRB #: 15–1797) approved this study.

### Procedure

**Fig. 1:**
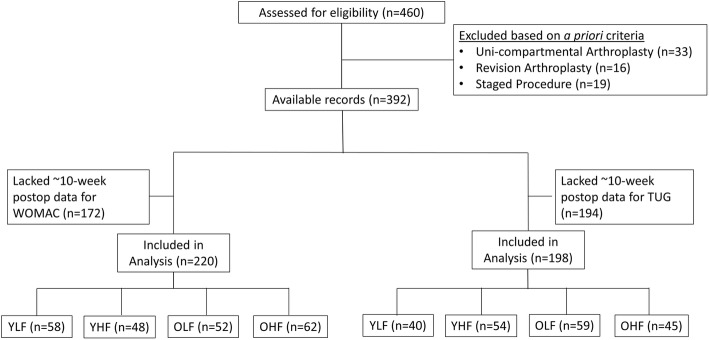
CONSORT flow diagram. Abbreviation: WOMAC, Western Ontario and McMaster Universities Osteoarthritis Index; TUG, Timed Up and Go; YLF, younger low functioning; YHF, younger high functioning; OLF, older low functioning; OHF, older high functioning; postop, postoperative

To fully capture the construct “physical function,” we operationally defined postoperative function two ways: 1) physical performance (TUG) and 2) self-reported function (WOMAC physical function subscale). Subgroups were defined based on 1) age (median split: younger < 65 vs. older ≥ 65 years) and 2) a dichotomous division of postoperative TUG (median split: lower functioning ≥ 8.12 versus higher functioning < 8.12 s) and postoperative WOMAC physical function subscale (median split: lower functioning > 25% versus higher functioning ≤ 25%) scores (Fig. 1). The age cutoff of 65 years has shown to be an appropriate indicator for defining relatively older (≥ 65 years) and younger (< 65 years) patients with TKA [3]. The TUG and WOMAC cutoffs were defined based on clinical and statistical insight as no study has examined categorization of lower and higher functioning subgroups within the TKA patient population. Thus, 4-group categorical variables were created to define: 1) younger lower functioning, 2) younger higher functioning, 3) older lower functioning and 4) older higher functioning, based separately for the TUG and WOMAC measures of physical function. Subgroup comparisons were based on patients’ functional measures collected at 10-weeks following surgery (mean, 67 days; range, 57 to 84 days). Patients at this recovery timepoint have typically 1) recovered from acute knee pain, 2) retained peak knee motion, 3) regained mobility and begun to experience a plateau in function [13, 16].

The TUG is a performance outcome measuring the time (seconds) taken for a person to rise from standard chair, walk 3 m, turn around, walk back to the chair and sit down [17]. Patients were instructed to complete the test as quickly, but as safely as possible. The WOMAC is a 24-item instrument, with three subscales [pain (5 items), stiffness (2 items), physical function (17 items)], measuring symptoms related to functional limitations in persons with knee pathology [18]. Scores for each subscale were converted to a percentage, with 100% corresponding to maximum functional limitation. The WOMAC physical function subscale was used to define subgroups for the purposes of this analysis. The TUG [17, 19–21] and WOMAC [18, 22–24] measures have both shown to be reliable and valid instruments, and are commonly utilized in the TKA population [18, 21].

### Baseline characteristics and outcome assessments

Preoperative characteristics collected immediately before surgery (baseline) were included for outcome assessment and defined within four domains: health status, psychological status, physical performance and knee pain intensity. Each domain included two measures that provided context for the preoperative characteristics. *Health status* was described by body mass index and the Functional Comorbidities Index as indicators of general health [25]. The body mass index was based on weight (kg) and height (m) metrics obtained at baseline. The Functional Comorbidities Index is a sum of 18 self-reported comorbid conditions ranging from 0 to 18 (higher scores indicating greater comorbidity). Both body mass index and comorbidity status have been associated with functional outcomes in previous knee osteoarthritis phenotyping analyses as well as TKA prognostic analyses [26–28]. *Psychological status* was assessed using the Patient Health Questionnaire [29] as a measure of depression and the Coping Strategies Questionnaire-Catastrophizing Subscale [30] as a measure of emotional distress related to disability. The Patient Health Questionnaire (2-item) score was tabulated based on a 0–6 scale (higher scores indicate greater degree of depression). The Coping Strategies Questionnaire-Catastrophizing Subscale (2-item) was tabulated based on a 0–12 scale (higher scores indicate greater pain catastrophizing) [31]. Preoperative depression and pain catastrophizing are linked with poorer outcomes following TKA surgery [10, 32]. *Physical performance* was described by maximum isometric knee extensor strength and gait speed. Knee extensor strength was measured with participants placed in a seated position and the surgical knee constrained at 60-degree of knee flexion [33]. An average of three maximum isometric contractions using a handheld dynamometer (Lafayette Instrument Corp, Lafayette, IN, USA) was used for analysis and normalized to body mass (kg) [34]. Gait speed was measured using the 4-m walk test and an average of two self-selected trials were used for analysis [35]. Preoperative knee extensor strength is strongly associated with postoperative strength and functional outcomes, and usual gait speed is an important indicator of overall physical health [36, 37]. *Pain intensity* was described by numeric pain rating scale of the contralateral knee and WOMAC pain subscale of the operative knee [24, 38]. The numeric pain rating score was based on a 11-point scale (0 = no pain and 10 = worst possible pain imaginable) and used as a proxy for contralateral knee pain intensity [38]. The WOMAC pain subscale (5 items) score was tabulated based on a 0–4 scale (lower scores indicate lower levels of pain) and used as a proxy for surgical knee pain intensity [39].

### Statistical analysis

General linear models were used to examine subgroup differences between the younger lower functioning subgroup and other remaining subgroups, (defined according to both TUG and WOMAC physical function subscale scores) for the following preoperative domains: health status (BMI and Functional Comorbidities Index), psychological status (Patient Health Questionnaire and Coping Strategies Questionnaire-Catastrophizing Subscale), physical performance (knee extensor strength and gait speed) and pain intensity (contralateral knee pain and WOMAC pain subscale), controlling for sex and timing of postoperative assessment. To minimize the number of contrasts performed, the younger lower functioning subgroup was considered the reference group for all analyses. Additionally, the *p*-values for all pairwise tests were adjusted using Tukey-Ciminera-Heyse multiple comparison procedure [40]. Alpha level to test for statistical significance was set at *p* ≤ 0.05. Missing data were imputed using the method of multiple multivariate imputation described by van Buuren et al. [41] Missing data was assumed to be missing at random, therefore the imputations could be improved by assuming they were correlated with other variables. Multiple multivariate imputation via chained equations were applied, as implemented in the STATA Version 14.1 statistical software (College Station, TX, USA), using 30 imputed sets that were combined using Rubin’s rules [42, 43].

## Results

Of the initial 460 TKA patient records available, 68 records were excluded based on a priori selection criteria, leaving 392 eligible patient records. A proportion of the remaining records could not be examined because they lacked 10-week postoperative functional assessment. Thus, for groups defined on TUG scores, 192 records were available for analysis, whereas for groups defined on WOMAC physical function subscale scores, 220 records were available for analysis (Fig. 1). Based on subgroup classifications, a 4-group categorical variable was created for TUG scores: younger lower functioning (*n* = 40), younger higher functioning (*n* = 54), older lower functioning (*n* = 59), older higher functioning (*n* = 45) and WOMAC physical function subscale scores: younger lower functioning (*n* = 58), younger higher functioning (*n* = 48), older lower functioning (*n* = 52), older higher functioning (*n* = 62). Descriptive statistics were computed for demographic characteristics and preoperative functional measures (Table 1).

**Table 1:** Descriptive characteristics of participants based on subgrouping at baseline

Variable/Subgroups	YLF	YHF	OLF	OHF
Postop WOMAC	(*n* = 58)	(*n* = 48)	(*n* = 52)	(*n* = 62)
Age, y	56.7 (5.5)	57.8 (5.7)	72.3 (5.1)	73.0 (5.7)
Sex, n (% male)	20 (34.5)	21 (43.8)	17 (32.7)	31 (50.0)
Preop TUG score	11.3 (3.0)	9.1 (2.8)	13.7 (6.8)	11.3 (5.3)
Preop WOMAC score	0.62 (0.17)	0.50 (0.18)	0.53 (0.16)	0.47 (0.15)
Postop TUG	(*n* = 40)	(*n* = 54)	(*n* = 59)	(*n* = 45)
Age, y	57.4 (6.2)	57.8 (5.7)	72.3 (5.1)	73.0 (5.7)
Sex, n (% male)	8 (20.0)	30 (55.6)	21 (35.6)	25 (55.6)
Preop TUG score	11.8 (3.7)	8.6 (2.2)	16.6 (10.0)	10.1 (4.9)
Preop WOMAC score	0.59 (0.16)	0.48 (0.19)	0.54 (0.18)	0.46 (0.17)

*Note:* Values represented as mean (SD), unless otherwise stated. *YLF* Young and low functioning, *YHG* Young and high functioning, *OLF* Old and low functioning, *OHF* Old and high functioning, *TUG* Timed up and go, *WOMAC* Western Ontario and McMaster Universities Osteoarthritis Index, *Postop* postoperative, *Preop* preoperative

**Fig. 2:**
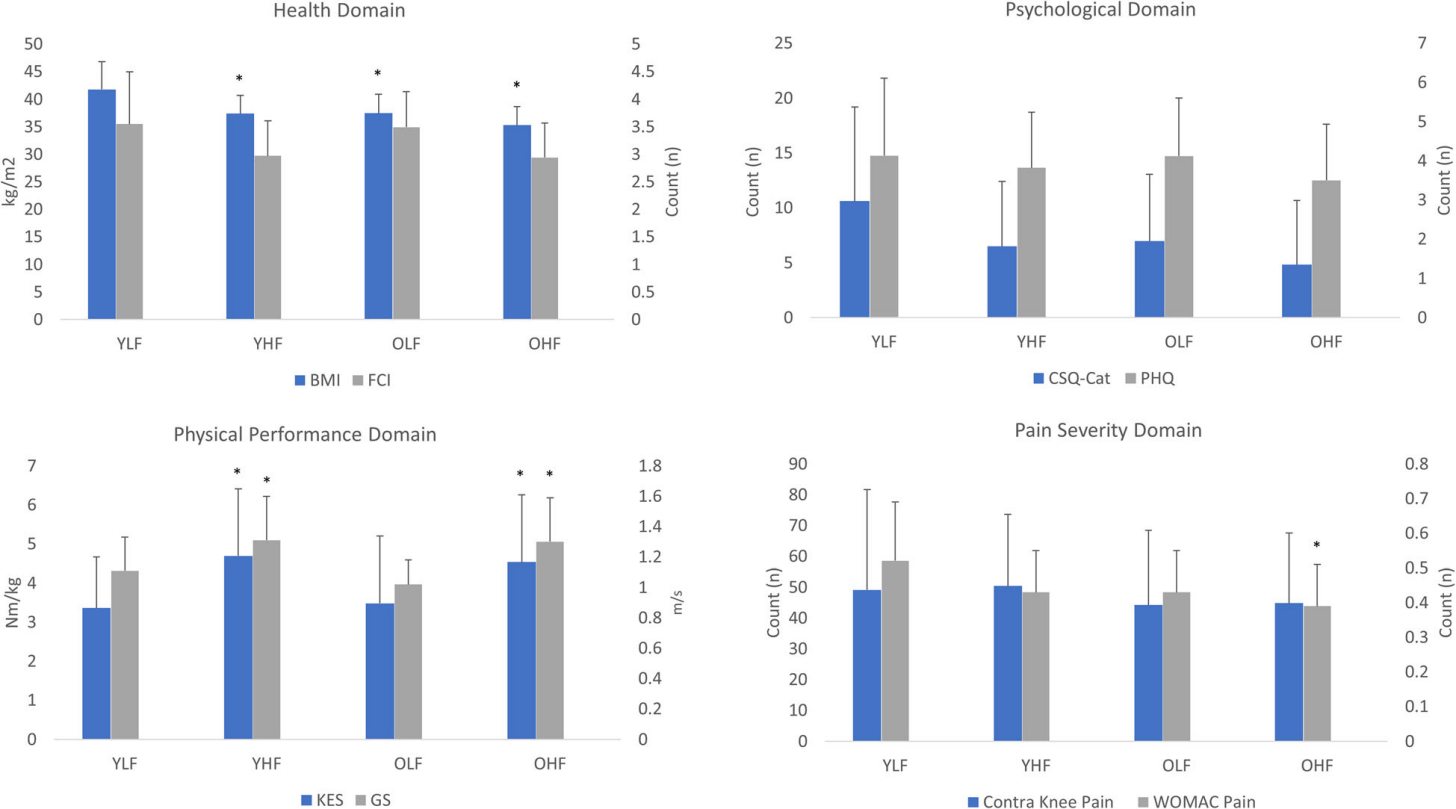
Graphic display of Timed Up and Go (TUG) subgroup results. Values represented as mean (standard error). Abbreviation: BMI, body mass index; FCI, functional comorbidities index; PHQ, patient health questionnaire; CSQ-Cat, Coping Strategies Questionnaire-Catastrophizing Subscale; KES, knee extensor strength; GS, gait speed; Contra, contralateral

Compared to the older higher functioning subgroup using the TUG, the younger lower functioning subgroup demonstrated significantly lower preoperative knee extensor strength [mean difference (MD), − 1.02 ± 0.47 Nm/kg; t-statistic, − 2.16; 95% CI, − 1.96, − 0.08; *p* = 0.04], slower preoperative gait speed (MD, 0.21 ± 0.08 m/s; t-statistic, 2.65; 95% CI, 0.05, 0.37; *p* = 0.05), higher body mass index (MD, 8.3 ± 1.6 kg/m^2^; t-statistic, 5.05; 95% CI, 5.05, 11.54; *p* < 0.01) and higher preoperative WOMAC pain subscale scores (MD, 0.14 ± 0.05; t-statistic, 2.88; 95% CI, 0.04, 0.24; *p* = 0.03) (Fig. 2). Compared to the younger higher functioning subgroup using the TUG, the younger lower functioning subgroup demonstrated significantly lower preoperative knee extensor strength (MD, − 1.65 ± 0.47 Nm/kg; t-statistic, 3.53; 95% CI, − 2.58, − 0.72; *p* = 0.02), slower preoperative gait speed (MD, 0.20 ± 0.08 m/s; t-statistic, 2.58; 95% CI, 0.05, 0.36; *p* = 0.03), and higher body mass index (MD, 6.1 ± 1.6 kg/m^2^; t-statistic, 3.92; 95% CI, 3.04, 9.20; *p* = 0.05). Compared to the older lower functioning subgroup using the TUG, the younger lower functioning subgroup demonstrated significantly higher body mass index (MD, 6.1 ± 1.6 kg/m^2^; t-statistic, 3.92; 95% CI, 3.03, 9.20; *p* = 0.03). After correction for multiple comparisons, no other significant differences were observed in any other outcome measure between any of the subgroups.

**Fig. 3:**
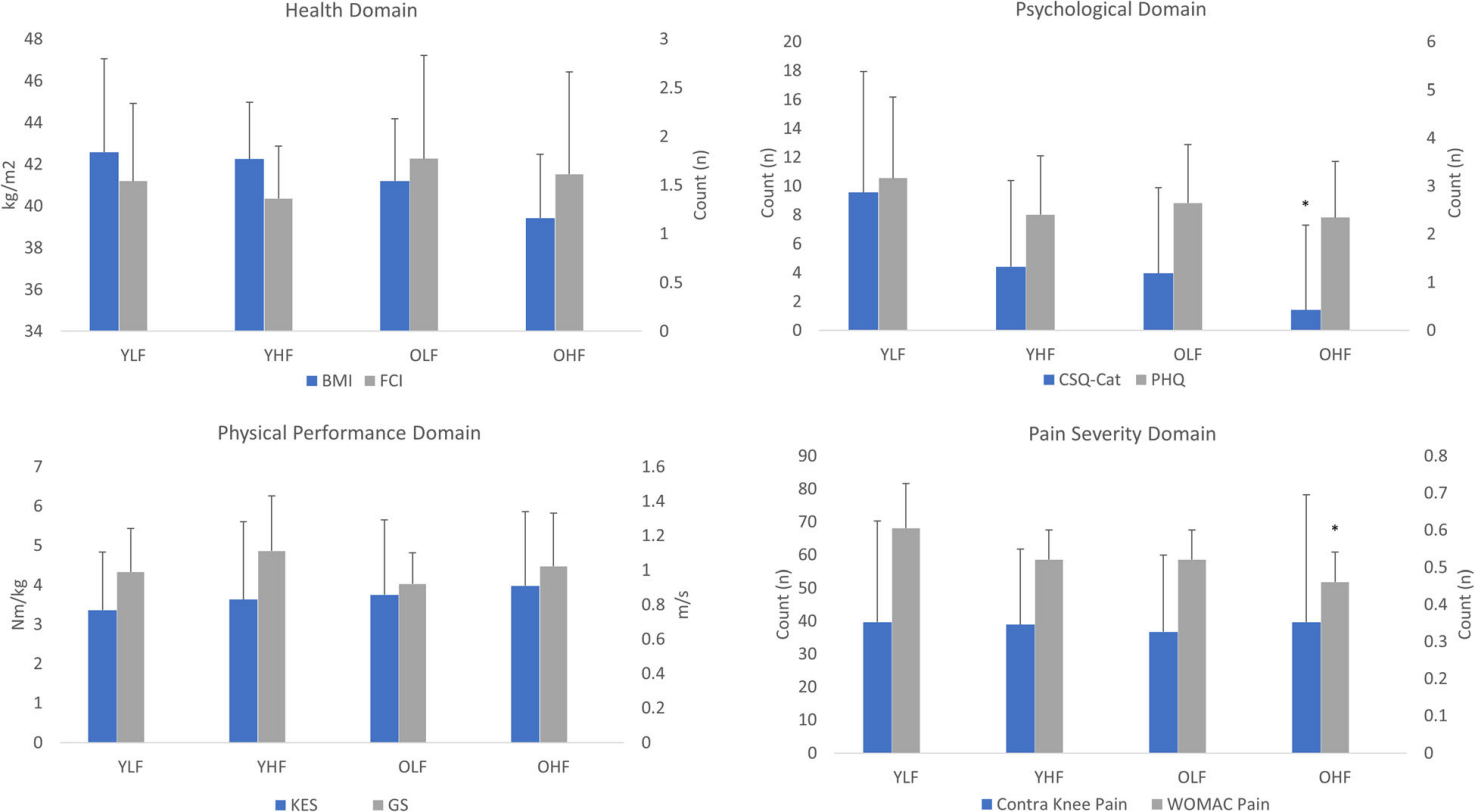
Graphic display of Western Ontario and McMaster Universities Osteoarthritis Index (WOMAC) subgroup results. Values represented as mean (standard error)*.* Abbreviation: BMI, body mass index; FCI, functional comorbidities index; PHQ, patient health questionnaire; CSQ-Cat, Coping Strategies Questionnaire-Catastrophizing Subscale; KES, knee extensor strength; GS, gait speed; Contra, contralateral

Compared to the older higher functioning subgroup using the WOMAC physical function subscale score, the younger lower functioning subgroup demonstrated significantly worse WOMAC pain subscale scores (MD, 0.15 ± 0.04; t-statistic, 3.68; 95% CI, 0.06, 0.23; *p* < 0.01) and Coping Strategies Questionnaire-Catastrophizing Subscale scores (MD, 7.8 ± 2.6; t-statistic, 3.02; 95% CI, 2.66, 12.89; *p* = 0.02) (Fig. 3). After correction for multiple comparisons, no other significant differences were observed in any other outcome measure between any of the subgroups.

## Discussion

In this observational study, we evaluated relatively young lower functioning patients following TKA surgery, to determine whether and how this group differed from those with a more typical postoperative recovery response. The principal findings were: [1] younger lower functioning patients, using postoperative physical performance (TUG) as a measure of functional recovery, demonstrated significantly higher preoperative body mass index measures compared to all other subgroups [2]; younger lower functioning patients, using postoperative physical performance (TUG), demonstrated worse preoperative knee extensor strength and slower gait speed compared to both the younger and older higher functioning subgroups [3]; younger lower functioning patients, using postoperative physical performance (TUG), demonstrated higher preoperative WOMAC pain subscale scores compared to the older higher functioning subgroup [4]; younger lower functioning patients, using postoperative self-report (WOMAC physical function subscale) as a measure of functional recovery, demonstrated significantly higher WOMAC pain subscale and Coping Strategies Questionnaire-Catastrophizing Subscale scores compared to the older higher functioning subgroup.

Identifying preoperative characteristics associated with postoperative challenges is important to effectively identify more appropriate surgical candidates and assist in screening those who are less optimal for functional recovery. Our results suggest several factors that could be important in shaping postoperative rehabilitation strategies. First, the younger lower functioning subgroup using the TUG demonstrated poorer preoperative knee extensor strength and gait speed relative to higher functioning counterparts. These factors have consistently been identified as predictors of postoperative functioning across multiple studies [44, 45]. Preoperative knee extensor strength is not only a strong predictor of early functional performance [13], but also accounts for a large portion of the variance explained in diminished walking and stair climbing ability at 1 year [36]. Our findings showed the younger and older higher functioning subgroups presented with greater than one body unit difference in knee extensor strength relative to younger lower functioning subgroup when classified using the TUG. These findings are clinically relevant showing the importance of identifying muscular deficits preoperatively as a metric of postoperative functional recovery and was consistent despite the age categorization. Additionally, slower gait speed has also shown to be an important predictor of functional decline and increased fall risk in older adults, and has been advocated as a sixth vital sign by practicing clinicians [46–48]. Our findings showed a clinically relevant faster preoperative gait speed in both the younger (0.20 m/s) and older higher functioning (0.21 m/s) relative to the younger lower functioning subgroup when using the TUG, indicating velocity as an important metric on postoperative physical recovery that was consistent between functional subgrouping, despite age categorization. Rehabilitation strategies commonly recognize these factors and seek to target functional and strength deficits to maximize recovery [49]. Investigations have also shown that preoperative rehabilitation strategies can effectively improve in areas of muscle and physical performance, ultimately equating to increased functional outcomes postoperatively [50–53]. Our results speak to the importance of such efforts, as the younger lower functioning subgroup is likely to continue to increase in prevalence as surgical indications expand.

Secondly, higher preoperative body mass index was observed in the younger lower functioning subgroup using the TUG relative to all other subgroups. Several studies have shown similar findings of body mass indexes > 30 kg/m^2^ to be associated with increased postoperative knee pain, higher physical inactivity, lower functional performance and greater postoperative complications [54, 55]. Targeted preoperative weight loss management interventions have shown inconsistent improvements in postoperative outcomes for patients undergoing TKA [56]. Additionally, weight loss with or without bariatric surgery has also shown marginal clinical outcomes and complication rate reductions following subsequent arthroplasty surgery [57, 58]. Knowing overweight and obese patients are still in need of surgical intervention, it is important to educate patients on the impact body mass index has on postoperative physical performance in those contemplating surgery. Further investigation is needed to better understand the impact age and different weight classes have on postoperative outcomes, while promoting healthy lifestyle choices with increased physical activity postoperatively.

Thirdly, greater preoperative pain perception and pain-related limitations in functional ability were observed in the younger lower functioning subgroup compared to older higher functioning subgroup using the WOMAC physical function subscale. These findings were consistent with further findings showing preoperative pain-related limitations were greater in the younger lower functioning subgroup when compared to the older higher functioning subgroup using the TUG. Thus, preoperative pain may be a determinant of postoperative performance and self-report function in younger patients relative to older higher functioning peers. Preoperative pain severity has previously been identified as a strong predictor of postoperative physical performance among older adults [59]. However, more intense pain experiences have been shown to be related with neurophysiological processes associated with pain modulation and are more common in younger adults [60–62]. Additionally, younger adults typically present with less radiographic evidence of arthritis preoperatively compared to older peers [63–65]. Data has further shown lack of radiographic severity has an inverse relationship on function, showing poorer physical performance and pain related outcomes postoperatively [64, 65]. Alternative mechanisms of pain perception and disability, unrelated to arthritic changes of the knee, may affect surgical and non-surgical care decisions among younger patients. Pain coping strategies might be an important treatment strategy in these patients [66]. Physical therapists may be optimally positioned to effectively intervene with these coping skills as they treat a large volume of these patients prior to and following surgery [67]. Young patients undergoing TKA may also exhibit greater complexity than their older counterparts, as different expectations, comorbidity risks, psychosocial and mental health concerns potentially influence postoperative functional outcomes. Further research is needed in this area with a more comprehensive evaluation of the younger patient to be considered prior to surgery to better understand predictive characteristics that effect functional recovery.

Fourthly, our findings indicate a disconnect between self-report and physical performance-based subgrouping, as they pertain to preoperative characteristics influence following TKA. Younger lower functioning patients showed greater disparity across groups when defined by the TUG as compared to the WOMAC physical function classification. Preoperative characteristics appear to be more predictive of postoperative physical performance measures as compared to a self-report metric. Poor concurrent validity between self-report and physical performance measures have been reported following TKA [13]. However, both measures are needed to understand the full scope of physical function recovery in this patient population. Exclusively using self-report measures tends to overestimate the short- and long-term changes in physical function following TKA [13], although this metric is more frequently reported relative to physical performance. Patients with TKA commonly perceive improved physical function during the early postoperative period, while further showing significant impairments in objective physical performance [13, 15]. Both measures are necessary to fully understand the change in functional recovery as perception and performance-based assessments provide complimentary, but different indicators of impairment [13]. These findings could have important influence on functional recovery and inferences in clinical decision making. If postoperative recovery is based primarily on the patient’s self-report of functional recovery, then important activity limitations of the patient may go unrecognized and untreated. However, understanding the influence preoperative characteristics have on postoperative recovery could help inform prognosis and shape treatment strategies to maximize outcomes in this problematic but growing patient demographic.

This study should be interpreted considering its strengths and limitations. First, we sampled a relatively large heterogenous group of patients with TKA that were unbounded by extensive eligibility requirements. This pragmatic approach more realistically represents a general clinical population and is a strength of our study. However, a more controlled study design minimizing potential confounders could be considered in future investigations. Second, we evaluated postoperative function at approximately 10-weeks following surgery as this time frame includes the typical period of rehabilitation. Longer-term data collection may be helpful to better represent a functional recovery plateau, which may occur 6 months or more following surgery. Third, the STROBE (Strengthening The Reporting of OBservational Studies in Epidemiology) checklist was used to facilitate critical appraisal and interpretation of the study. Fourth, we classified the subgroups based on relatively arbitrary group definitions (a median split); further work could explore data-driven subgrouping approaches that may be better able to examine prevalence of a younger lower functioning subgroup. By subgrouping the patient data, power is reduced, and despite our robust sample size future work will benefit from larger samples and statistical approaches that allow for continuous representation of the data. However, a subgrouping approach was used as an initial examination of this question and to improve clinical interpretability.

## Conclusion

Poorer preoperative physical performance and pain severity domains appear to have the largest influence on early postoperative TKA recovery in younger and lower functioning patients relative to both older and younger higher functioning patients. These findings indicate modifiable characteristics such as body mass index, knee extensor strength, walking speed and pain management strategies may all influence the postoperative functional recovery and expectations for younger, lower functioning patients undergoing TKA.

## Data Availability

The datasets used and/or analyzed during the current study are available from the corresponding author on reasonable request.

## Abbreviations

TKA: Total knee arthroplasty
TUG: Timed Up and Go
WOMAC: Western Ontario and McMaster Universities Osteoarthritis Index
